# Blastomycosis-Associated Hospitalizations, United States, 2010–2020

**DOI:** 10.3390/jof9090867

**Published:** 2023-08-22

**Authors:** Kaitlin Benedict, Ian Hennessee, Jeremy A. W. Gold, Dallas J. Smith, Samantha Williams, Mitsuru Toda

**Affiliations:** 1 Mycotic Diseases Branch, Division of Foodborne, Waterborne and Environmental Diseases, Centers for Disease Control and Prevention, Atlanta, GA 30329, USA; xye0@cdc.gov (I.H.); leo3@cdc.gov (J.A.W.G.); rhq8@cdc.gov (D.J.S.); pog3@cdc.gov (S.W.); nrk7@cdc.gov (M.T.); 2 Epidemic Intelligence Service, Centers for Disease Control and Prevention, Atlanta, GA 30329, USA

**Keywords:** blastomycosis, hospitalization, epidemiology, mortality, United States

## Abstract

Background: Blastomycosis is an environmentally acquired fungal disease that can cause severe illness, with approximately 65% of reported cases requiring hospitalization. Recent trends in blastomycosis-associated hospitalizations in the United States have not been described. Methods: We analyzed hospital discharge data from the Healthcare Cost and Utilization Project (HCUP) National (Nationwide) Inpatient Sample. We calculated hospitalization rates per 100,000 population using U.S. census data and examined factors associated with in-hospital mortality. Results: An estimated 11,776 blastomycosis-associated hospitalizations occurred during 2010–2020 (average yearly rate 0.3 per 100,000 persons), with no apparent temporal trend. Rates were consistently highest among persons ≥65 years old and males. In-hospital death occurred in 7.9% and approximately doubled from 3.9% in 2010 to 8.5% in 2020. Older age, chronic obstructive pulmonary disease, and malignancy were associated with mortality. Conclusions: Blastomycosis-associated hospitalizations can result in poor outcomes, underscoring the continued need for attention to early detection and treatment of blastomycosis and monitoring of disease trends.

## 1. Introduction

Blastomycosis is a fungal disease caused primarily by inhalation of *Blastomyces dermatitidis* and *Blastomyces gilchristii* spores. In the United States, these fungi live in the environment mainly in the midwestern, south central, and southeastern states, though infections have also been acquired outside these areas, suggesting that the geographic range is broader than is generally believed [1,2,3]. A newly described species, *Blastomyces helicus*, has also caused infections in humans and animals in the western regions of Canada and the United States [4]. However, the precise ecologic niche of *Blastomyces* is poorly understood. Although cases are usually sporadic, large outbreaks of blastomycosis can also occur [5,6].

Approximately 65% of patients with blastomycosis cases reported to public health authorities require hospitalization [1]. However, public health surveillance for blastomycosis is limited to only five states (Arkansas, Louisiana, Michigan, Minnesota, and Wisconsin), so other data sources are essential for monitoring national trends. A previous analysis of hospital discharge data examined rates of blastomycosis-associated hospitalizations during 2000–2011 and found substantial increases in two of the five states (Illinois and Kentucky) with the highest incidence. Recent trends have not been described [7]. In addition, a deeper understanding of factors contributing to increased risk of mortality among blastomycosis patients is needed [8,9].

To understand recent trends in blastomycosis-associated hospitalizations and examine factors associated with in-hospital mortality among blastomycosis patients, we analyzed 2010–2020 data from the Healthcare Cost and Utilization Project (HCUP), a set of databases sponsored by the Agency for Healthcare Research and Quality.

## 2. Materials and Methods

The HCUP National (referred to as “Nationwide” before 2012) Inpatient Sample (NIS) is the largest publicly available all-payer database of hospital inpatient stays in the United States [10]. Starting in 2012, the NIS represents a 20% stratified sample of all discharges from U.S. community hospitals, excluding rehabilitation and long-term acute care hospitals; before 2012, the NIS contained all discharges from a sample of participating hospitals. To enable calculation of national estimates, discharge-level weights are assigned based on the hospital’s U.S. Census division, ownership, urban/rural location, teaching status, and number of beds. The unweighted NIS contains data on approximately 7 million inpatient stays each year, which translates to approximately 35 million inpatient stays when weighted.

In the United States, medical coding transitioned from the International Classification of Diseases, Ninth Revision, Clinical Modification (ICD-9-CM) codes to the International Classification of Diseases, Tenth Revision, Clinical Modification (ICD-10-CM) codes on 1 October 2015. The NIS contains up to 40 diagnosis codes per discharge record in 2020 (fewer for earlier data years). We identified blastomycosis-associated hospitalizations and concurrent conditions using selected ICD-9-CM and ICD-10-CM codes listed anywhere on the discharge record (Supplemental Table S1). Information about clinical forms of blastomycosis (e.g., pulmonary, cutaneous, disseminated) is available only in the ICD-10-CM coding scheme.

We obtained yearly national estimates of blastomycosis-associated hospitalizations using the HCUP-supplied discharge weights and examined trends by age group, sex, Census region (https://www2.census.gov/geo/pdfs/maps-data/maps/reference/us_regdiv.pdf, accessed on 8 August 2023), season of hospital admission, clinical form of blastomycosis, and presence of selected concurrent conditions using SAS 9.4 (SAS Institute, Cary, NC, USA) survey procedures. Overall rates and age-, sex-, and region-specific rates were calculated using population estimates from the U.S. Census Bureau [11]. We also evaluated demographic, socioeconomic, and medical factors associated with in-hospital mortality among blastomycosis-associated hospitalizations using Rao–Scott chi-square goodness-of-fit tests for categorical variables and linear regression analysis to compare domain means for continuous variables (α = 0.05). We used a weighted least-squares technique to test for linear trends in annual mortality rates [12]. This activity was reviewed by CDC and was conducted consistent with applicable federal law and CDC policy (See e.g.: 45 C.F.R. part 46, 21 C.F.R. part 56; 42 U.S.C. §241(d); 5 U.S.C. §552a; 44 U.S.C. §3501 et seq).

## 3. Results

**Figure 1:**
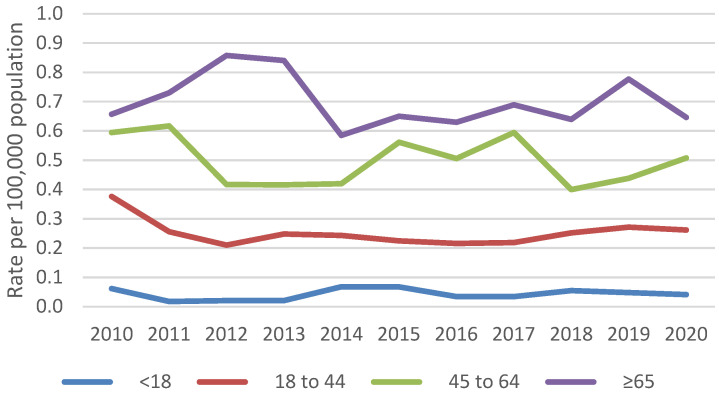
Age-specific blastomycosis-associated hospitalization rates per 100,000 population, 2010–2020.

**Figure 2:**
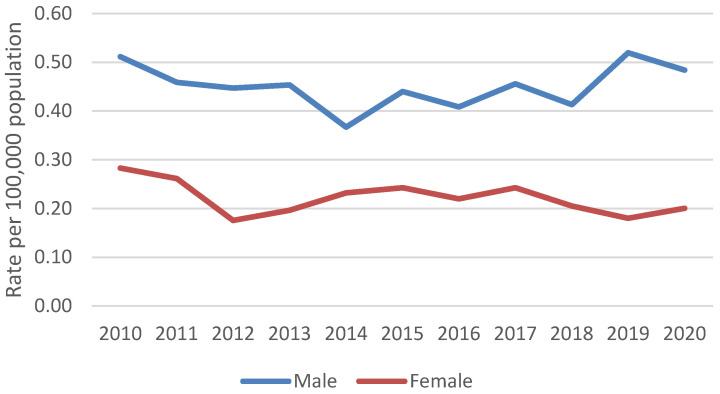
Sex-specific blastomycosis-associated hospitalization rates per 100,000 population, 2010–2020.

**Figure 3:**
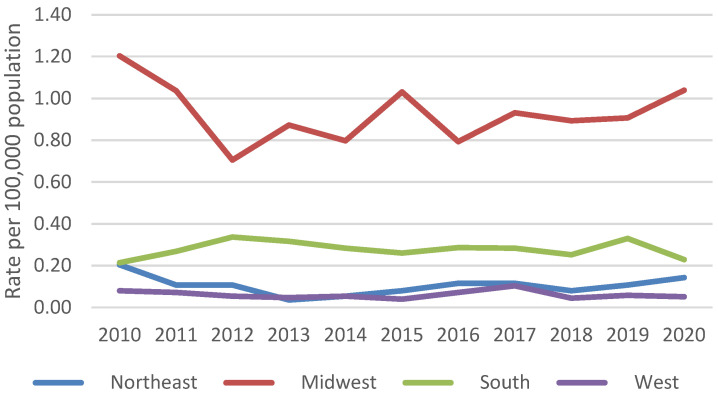
Regional blastomycosis-associated hospitalization rates per 100,000 population, 2010–2020.

**Figure 4:**
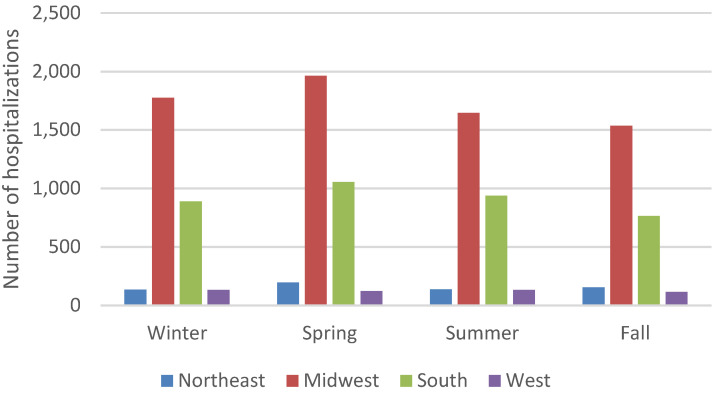
Blastomycosis-associated hospitalizations by admission season and region, 2010–2020.

An estimated 11,776 blastomycosis-associated hospitalizations occurred during 2010–2020. The average yearly rate was 0.3 hospitalizations per 100,000 persons. Yearly rates were consistently highest among persons ≥65 years old (average 0.7 per 100,000 persons) (Figure 1) and males (average 0.5 per 100,000 persons) (Figure 2). Most blastomycosis-associated hospitalizations occurred in the Midwest (58.8%, average rate 0.9 per 100,000 persons) and the South (31.4%, average rate 0.3 per 100,000 persons) (Figure 3). No temporal trends in overall blastomycosis-associated hospitalization rates or in age-, sex-, or region-specific rates were observed. By season, 29% of hospital admissions occurred in spring, 25% in winter, 24% in summer, and 22% in fall, with no clear seasonal differences by region (Figure 4).

**Figure 5:**
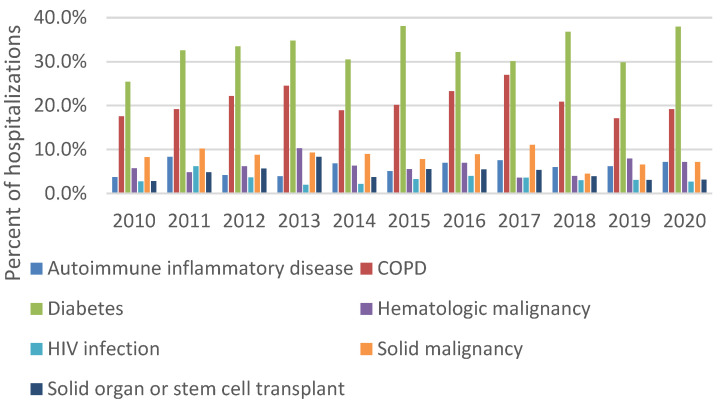
Percent of blastomycosis-associated hospitalizations with selected concurrent conditions, 2010–2020.

Blastomycosis was listed as the primary discharge diagnosis in 29.3% of all blastomycosis-associated hospitalizations. Diabetes (30.5%) and chronic obstructive pulmonary disease (COPD) (20.8%) were the most common selected concurrent conditions (Figure 5). Among 5595 hospitalizations with information about clinical forms of blastomycosis, 53.1% had pulmonary blastomycosis, 15.5% had disseminated blastomycosis, 5.3% had cutaneous blastomycosis, and 30.9% had “other or unspecified” forms listed. No temporal trends in concurrent conditions or clinical forms of blastomycosis were observed.

**Figure 6:**
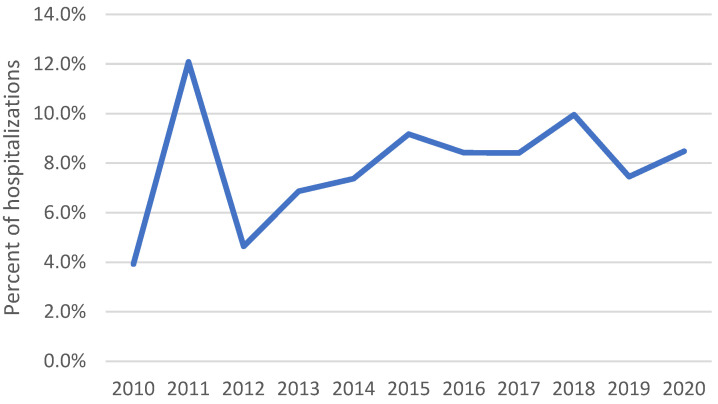
Percent of blastomycosis-associated hospitalizations with in-hospital mortality, 2010–2020.

Mean hospitalization length was 9.9 days (standard error 0.25 days) (Table 1). In-hospital death occurred in 7.9% of all blastomycosis-associated hospitalizations. The in-hospital mortality rate was 3.9% in 2010, peaked at 12.1% in 2011, and then increased significantly from 4.6% in 2012 to 8.5% in 2020 (*p* = 0.012) (Figure 6). Compared with patients who survived to discharge, those who died were older (61.8 vs. 53.0 years, *p* < 0.001) and more frequently had Medicare insurance (56.8% vs. 40.9%), COPD (27.6% vs. 20.3%, *p* = 0.020), hematologic malignancy (10.8% vs. 5.8%, *p* = 0.008), and solid malignancy (15.4% vs. 7.8%, *p* < 0.001).

## 4. Discussion

This analysis provides an update to the epidemiologic description of blastomycosis throughout the United States. Our results confirm that blastomycosis-associated hospitalizations remain relatively uncommon nationally (0.3 per 100,000 persons), which is consistent with the estimated incidence of 0.8 cases per 100,000 persons in public health surveillance data [1]. A major limitation of our study is the inability to conduct state-level analyses with the HCUP NIS, particularly because the previous report of blastomycosis-associated hospitalizations found substantial variations in state-specific trends over time [7]. However, we did observe higher rates of blastomycosis-associated hospitalizations in the Midwest and the South, aligning with the broad environmental distribution of *Blastomyces*. In addition, the occurrence of hospitalizations in other regions indicates that the geographic distribution of blastomycosis might be broader than previously recognized [2,3], and it is important for healthcare providers to note the potential for travel-associated blastomycosis cases [1]. Therefore, there is a need for ongoing awareness of blastomycosis throughout the country.

We did not observe changes over time in blastomycosis-associated hospitalization rates among specific demographic groups. The predominance of blastomycosis among males is well-described and might be related to biologic differences or occupational exposures [1]. However, previous studies have found conflicting results about the risk of hospitalization and mortality by sex [5,7,13], so further work in this area could be useful. The higher rate of blastomycosis-associated hospitalizations among older adults is not surprising and is consistent with prior studies [5,7]. In addition, the association between payer status and mortality probably reflects the age distribution of these patients.

Among the selected concurrent conditions examined in this analysis, the two most common were diabetes (30.5%) and COPD (20.8%); these proportions were higher than in a study of all patients with blastomycosis at a hospital network in Wisconsin and Minnesota (diabetes, 23% and COPD, 9%) [14] and in Minnesota public health surveillance data (diabetes, 17%) [6], but similar to the proportions among patients with blastomycosis at a tertiary care center in Kentucky (diabetes, 30% and COPD, 25%) [15], indicating that these conditions may be associated with more severe blastomycosis [6,16]. Our results did not reveal substantial temporal changes in the proportions of blastomycosis patients with certain concurrent conditions. Because existing public health surveillance for blastomycosis does not consistently capture information about concurrent conditions, hospital discharge data remain a valuable resource for monitoring potential changes in groups at higher risk of severe disease. Public health surveillance also does not always collect data about clinical forms of blastomycosis, so previous estimates that 25–40% of blastomycosis cases involve extrapulmonary involvement primarily come from geographically limited case series or outbreak investigations [16]. In general, the accuracy of ICD codes for identifying blastomycosis cases and subtypes is unknown and is therefore a limitation of this analysis, but our finding that nearly one-third of blastomycosis-associated hospitalizations were classified as “other or unspecified” forms of blastomycosis suggests a need for improved coding practices.

Strong seasonal patterns in blastomycosis-associated hospitalizations and clinical forms of blastomycosis were not evident at the regional or national level, with only a slightly larger proportion of hospitalization admissions in the spring (29%). In contrast, a study of blastomycosis patients from Manitoba and northwestern Ontario found that pulmonary disease was more common in the fall, whereas disseminated disease peaked in the spring [17]. U.S. surveillance data have shown either no clear seasonal pattern [5] or slightly more cases in the fall (29%) [1]. However, these studies used symptom onset date to classify cases by season, and many blastomycosis patients experience substantial delays in diagnosis [5,18], so using hospital admission date for analysis might obscure seasonal trends in exposure or symptom onset. Despite possible seasonal variations in exposure, healthcare providers should maintain suspicion for blastomycosis year-round given the potential for severe illness and poor patient outcomes.

Our results confirm that blastomycosis can be associated with substantial in-hospital mortality (8%), similar to mortality among cases reported through public health surveillance (9%) [1]. An increase in mortality over time was also observed among surveillance cases reported during 2007–2017 [5]; however, the reasons for the increase (as well as the peak in 2011) are unclear and do not appear to be related to changes in underlying conditions. Additional retrospective studies could be useful to capture other concurrent conditions not examined in this analysis and inform the observed mortality trend. The factors associated with mortality in this analysis (i.e., age, solid organ malignancy, hematologic malignancy, and COPD) generally align with previous studies [5,8,15]. Limitations include the inability to control for multiple predictors of mortality and that the NIS does not allow for de-duplication of multiple hospitalizations per patient.

Given the substantial morbidity and mortality associated with blastomycosis hospitalizations, reducing diagnostic delays is critical [16,18]. Expanded and strengthened public surveillance could generate more timely data for public health action.

## Figures and Tables

**Table 1:** Factors associated with in-hospital mortality among blastomycosis-associated hospitalizations, 2010 to 2020.

	Total	Died	Survived	
Characteristic	n = 11,776	n = 928	n = 10,848	*p*-Value
**Mean age, years (standard error [SE])**	53.6 (0.48)	61.8 (1.03)	53.0 (0.51)	<0.001
**Age group, years**				<0.001
<18	344 (2.9%)	not shown ^1^	not shown ^1^	
18 to 44	3212 (27.3%)	94 (10.1%)	3118 (28.7%)	
45 to 64	4552 (38.7%)	413 (44.5%)	4139 (38.2%)	
≥65	3668 (31.1%)	416 (44.8%)	3252 (30.0%)	
**Sex**				0.567
Male	7218 (61.3%)	598 (64.4%)	7218 (66.5%)	
Female	3959 (33.6%)	330 (35.6%)	3629 (33.5%)	
**Hospital region**				0.127
Northeast	643 (5.5%)	33 (3.6%)	610 (5.6%)	
Midwest	6921 (58.8%)	519 (55.9%)	6401 (59.0%)	
South	3702 (31.4%)	350 (37.7%)	3352 (30.9%)	
West	510 (4.3%)	25 (2.7%)	485 (4.5%)	
**Race/ethnicity (n = 10,994)**				0.079
White	7315 (66.5%)	627 (75.2%)	6687 (65.8%)	
Black	1859 (16.9%)	94 (11.3%)	1765 (17.4%)	
Hispanic	976 (8.9%)	53 (6.4%)	923 (9.1%)	
Other race/ethnicity	845 (7.7%)	60 (7.2%)	785 (7.7%)	
**Payer (n = 11,730)**				<0.001
Medicare	4941 (42.1%)	524 (56.8%)	4417 (40.9%)	
Medicaid	2009 (17.1%)	99 (10.7%)	1910 (17.7%)	
Private insurance	4363 (37.2%)	266 (28.8%)	4097 (37.9%)	
Other payer	418 (3.6%)	34 (3.7%)	384 (3.6%)	
**Income quartile for patient’s ZIP code (n = 11,562)**				0.575
0 to 25th percentile	4102 (35.5%)	337 (37.3%)	3765 (35.3%)	
26th to 50th percentile	3430 (29.7%)	292 (32.3%)	3138 (29.4%)	
51st to 75th percentile	2543 (22.0%)	182 (20.2%)	2361 (22.2%)	
76th to 100th percentile	1488 (12.9%)	93 (10.3%)	1395 (13.1%)	
**Admission season**				0.182
Winter	2937 (25.1%)	261 (28.3%)	2676 (24.8%)	
Spring	3343 (28.5%)	223 (24.2%)	3120 (28.9%)	
Summer	2860 (24.4%)	195 (21.1%)	2665 (24.7%)	
Fall	2577 (22.0%)	244 (26.4%)	2333 (21.6%)	
**Concurrent conditions**				
Autoimmune inflammatory disease	703 (6.0%)	54 (5.8%)	648 (6.0%)	0.954
Chronic obstructive pulmonary disease (COPD)	2454 (20.8%)	256 (27.6%)	2198 (20.3%)	0.020
Diabetes	3590 (30.5%)	298 (32.1%)	3652 (33.7%)	0.836
Hematologic malignancy	729 (6.2%)	100 (10.8%)	629 (5.8%)	0.008
HIV infection	387 (3.3%)	39 (4.2%)	348 (3.2%)	0.462
Solid malignancy	989 (8.4%)	143 (15.4%)	846 (7.8%)	<0.001
Solid organ or stem cell transplant	549 (4.7%)	25 (2.7%)	524 (4.8%)	0.178
**Mean length of hospitalization, days (SE)**	9.9 (0.25)	14.0 (0.93)	9.6 (0.25)	<0.001
**Clinical form of blastomycosis ^2^**	**n = 5595**	**n = 480**	**n = 5115**	
Pulmonary	2970 (53.1%)	300 (62.5%)	2670 (52.2%)	0.055
Cutaneous	295 (5.3%)	20 (4.2%)	275 (5.4%)	0.613
Disseminated	870 (15.5%)	80 (16.7%)	790 (15.4%)	0.754
Other or unspecified form	1730 (30.9%)	110 (22.9%)	1620 (31.7%)	0.076

^1^ Because of HCUP reporting restrictions, cells with ≤10 hospitalizations or cells that would enable calculation of a cell ≤10 are not displayed. ^2^ Among hospitalizations with ICD-10-CM data.

## Data Availability

Data are available from https://hcup-us.ahrq.gov/ (accessed on 8 August 2023).

## Supplementary Materials

The following supporting information can be downloaded at: https://www.mdpi.com/article/10.3390/jof9090867/s1, Table S1: ICD-9-CM and ICD-10-CM codes used to determine blastomycosis and other conditions of interest.
