# Supplementary material for: Blastomycosis-Associated Hospitalizations, United States, 2010–2020
Source: J Fungi (Basel). 2023 Aug 22;9(9):867. doi: 10.3390/jof9090867 (PMC10532734; doi:10.3390/jof9090867)
Supplement: Supplementary file 1 [file jof-09-00867-s001.zip › jof-2505400-supplementary.pdf]

Table S1. ICD-9-CM and ICD-10-CM codes used to determine blastomycosis and other conditions of interest.

Condition	ICD-9 Code(s)	ICD-10 Code(s)
Autoimmune inflammatory disease	714.0, 714.2, 555, 556, 696.0, 696.1, 696.8	K50–K51, L40, M02.3, M05–M06, M08, M33, M35.2, M45
Blastomycosis	116.0	B40
Pulmonary	n/a	B40.0, B40.1, B40.2
Cutaneous	n/a	B40.3
Disseminated	n/a	B40.7
Other or unspecified	n/a	B40.8, B40.9
Chronic obstructive pulmonary disease (COPD)	491, 492, 496	J41–J44
Diabetes	249, 250	E08–E13
Hematologic malignancy	200–208	C81–C95
HIV infection	042	B20, Z21
Solid malignancy	140–199, 235–239	C00–C81 (excluding C44)
Solid organ or stem cell transplant	V42 (excluding V42.3–V42.5)	Z94 (excluding Z94.5–Z94.7)
